# Trends in Esophageal Adenocarcinoma and Esophageal Squamous Cell Carcinoma Incidence in the United States from 1992 to 2019

**DOI:** 10.3390/cancers14246049

**Published:** 2022-12-08

**Authors:** Kyle S. Liu, Syed Ahsan Raza, Hashem B. El-Serag, Aaron P. Thrift

**Affiliations:** 1 Section of Epidemiology and Population Sciences, Department of Medicine, Baylor College of Medicine, Houston, TX 77030, USA; 2 Section of Gastroenterology and Hepatology, Department of Medicine, Baylor College of Medicine, Houston, TX 77030, USA; 3 Center for Innovations in Quality, Effectiveness and Safety (IQuESt), Michael E DeBakey Veterans Affairs Medical Center, Houston, TX 77021, USA; 4 Dan L Duncan Comprehensive Cancer Center, Baylor College of Medicine, Houston, TX 77030, USA

**Keywords:** esophageal cancer, incidence, birth cohort

## Abstract

**Simple Summary:**

The incidence of esophageal cancer overall has increased in the United States, driven by increasing rates of esophageal adenocarcinoma (EAC). However, whether rates of EAC are still rising is unclear. We examined trends in esophageal cancer overall and within important sub-groups of the population. We found that esophageal squamous cell carcinoma (ESCC) rates have been steadily declining, while EAC rates rose rapidly before stabilizing in 2000. The trend of decreasing incidence of ESCC was observed almost uniformly by age group, sex, and race/ethnicity, while trends in EAC rates varied across these sub-groups. A cohort effect for EAC was observed among people born during 1950, but EAC rates were stable across successive generations born between 1950 and 1985. Given the continued rising rates of known EAC risk factors, including obesity and gastroesophageal reflux disease, there is a need to continue monitoring trends for changes in incidence rates.

**Abstract:**

Background: Esophageal cancer (EC) incidence rates overall have declined in recent decades; however, the two main subtypes, esophageal adenocarcinoma (EAC) and esophageal squamous cell carcinoma (ESCC), show divergent secular trends. Methods: Age-adjusted EC incidence rates were calculated using data from the Surveillance Epidemiology and End Results (SEER) 12 Program. We examined secular trends from 1992 to 2019 overall and by age group, sex, race/ethnicity, tumor location, and SEER registry. Joinpoint regression was used to compute annual percent changes (APC) and average annual percent changes (AAPC). We used age-period-cohort models to examine the potential impact of period and birth cohort effects on trends. Results: Between 1992 and 2019, overall EC incidence rates declined by 0.54% annually (95% confidence interval [CI]: −0.75%, −0.33%). While ESCC rates declined linearly throughout the study period (AAPC = −2.85; 95%CI: −3.05%, −2.65%), EAC rates increased by over 5% annually from 1992 to 2000 (APC = 5.17; 95%CI: 3.28%, 7.10%), before stabilizing from 2000 to 2019 (APC = 0.22; 95%CI: −0.16%, 0.60%). Trends in ESCC and EAC varied by age group, sex, and race/ethnicity. Relative to ESCC rates among cohorts born circa 1950, the rates were 81% lower in cohorts born circa 1985 (rate ratio, 0.19; 95%CI: 0.04, 0.96). For EAC, rates have remained stable across successive birth cohorts since 1950. Conclusions: We observed linear declines in EC rates overall and for ESCC across age, sex, and race/ethnicity subgroups, but an inconsistent pattern for EAC. The trends in EAC cohorts born after 1955 were stable and suggest that EAC rates may have peaked in the U.S.

## 1. Introduction

Esophageal cancers (EC) continue to be a major contributor to the global cancer burden, with over 572,000 people diagnosed in 2018, representing 3.2% of all new cancer diagnoses worldwide [1]. Despite a reduction in overall EC incidence and mortality rates in the United States (U.S.), the change in the rates varied between two main subtypes, esophageal adenocarcinoma (EAC) and esophageal squamous cell carcinoma (ESCC). From the early 1970s to the late 2000s, the incidence of EAC increased dramatically in the U.S. However, the rate of increase has subsequently slowed, and rates appear to have peaked around 2005 [2]. Recent studies have suggested that EAC rates may still be increasing among young adults who are also presenting at more advanced stages with worse 5-year survival outcomes compared to patients who are older than 50 years at diagnosis [3]. Another study reported EAC trends according to sex and race/ethnicity, with increasing rates observed among non-Hispanic White (NHW) men aged <50 years, NHW women aged <70 years, as well as among Asian and Pacific Islander (API) men [4]. In contrast to EAC, incidence rates of ESCC have decreased in the U.S., but whether these rates are continuing a downward trend requires clarification [5].

In this study, we examined current trends in EC incidence using updated U.S. cancer registry data. Utilizing this population-based resource, with cases diagnosed from 1992 to 2019, we assessed secular trends in incidence rates overall and by age group, sex, race/ethnicity, and geographic regions for EC overall as well as the main EC subtypes, namely EAC and ESCC.

## 2. Methods

We used cancer incidence data from the Surveillance, Epidemiology, and End Results (SEER) 12 program. SEER is maintained by the National Institutes of Health and is a comprehensive, national database containing de-identified data for incident cancer cases in the U.S., including EC. We included incident primary EC cases aged ≥20 years and diagnosed between 1992 and 2019. We identified cases within the SEER 12 registries (Alaska Native Tumor Registry, Connecticut, Atlanta, Rural Georgia, San Francisco-Oakland, San Jose-Monterey, Hawaii, Iowa, Los Angeles, New Mexico, Seattle-Puget Sound, and Utah) using a combination of International Classification of Diseases for Oncology, 3rd edition (ICD-O-3) site codes (C15.0–C15.9) and ICD-O-3 histology codes (8050–8078, 8083–8084, 8140–8573). Histology codes 8140–8573 were used for EAC cases while codes 8050–8078 and 8083–8084 were used for ESCC.

### Statistical Analysis

SEER*Stat was used to compute annual age-standardized incidence rates of EC both overall and stratified by sex, race/ethnicity, and geography. We utilized the most recent SEER 12 release (15 April 2022) [6], which includes cancer cases from the registries previously included in SEER 13 except Detroit (data releases prior to 2022) [7,8]. Rates were standardized to the 2000 U.S. population using the direct method and are reported per 100,000 person-years. Corresponding 95% confidence intervals (CIs) were calculated using the modification described in Tiwari et al. [9]. We also examined age-specific EC rates based on defined age groups at the time of cancer diagnosis. Starting with the November 2021 data submission (data released 15 April 2022), race and ethnicity data were reported in five mutually exclusive categories: NHW, non-Hispanic Black (NHB), API, non-Hispanic American Indian/Alaska Native (AI/AN), and Hispanic [10]. In addition to EC incidence rates overall, we examined secular trends separately for EAC and ESCC.

We evaluated secular trends in EC incidence rates via the National Cancer Institute’s (NCI) Joinpoint program (version 4.9.1.0) (Bethesda, MD, USA), which tests whether an apparent change in trend is statistically significant using a Monte Carlo Permutation method [11]. We tested a single-line model (i.e., no joinpoints) and then assessed if more joinpoints should be added to the model based on their statistical significance. We allowed a maximum of two joinpoints with a minimum of two observations per segment [12]. The best joinpoint model was identified using log-transformed data. We obtained the annual percentage change (APC) in incidence rates over a single linear segment and the average annual percentage change (AAPC) over the entire study period for each joinpoint model. The 95% CIs were calculated using a normal approximation [13]. A parallelism test was used to examine whether the slopes of the change in trend between groups were similar in direction. A statistically significant *p*-value on this test indicates that the two trends in terms of AAPCs compared were statistically significantly different from each other [14]. All tests were two-sided with a statistical significance level of α = 0.05.

Finally, we used age-period-cohort models to search for patterns in secular incidence trends accounting for age at cancer diagnosis (age), year of cancer diagnosis (period), and year of birth (cohort). These models were fit using the NCI’s Age-Period-Cohort web tool (https://analysistools.cancer.gov/apc/, accessed on 30 June 2022), which provided estimates of net drifts (APC in expected age-adjusted rates over time), local drifts (APC in expected age-specific rates over time), and cohort rate ratios (ratio of age-specific rates in each birth cohort relative to the reference cohort). It also enables testing of equality of observed trends [15]. We used eleven 5-year age groups (30–34 years to 80–84 years), and five 5-year calendar periods (1995–1999 to 2015–2019). The calendar period of 2005–2009 and the birth cohort of 1950 were used as reference groups for comparisons.

## 3. Results

### 3.1. Overall Trends

**Figure 1:**
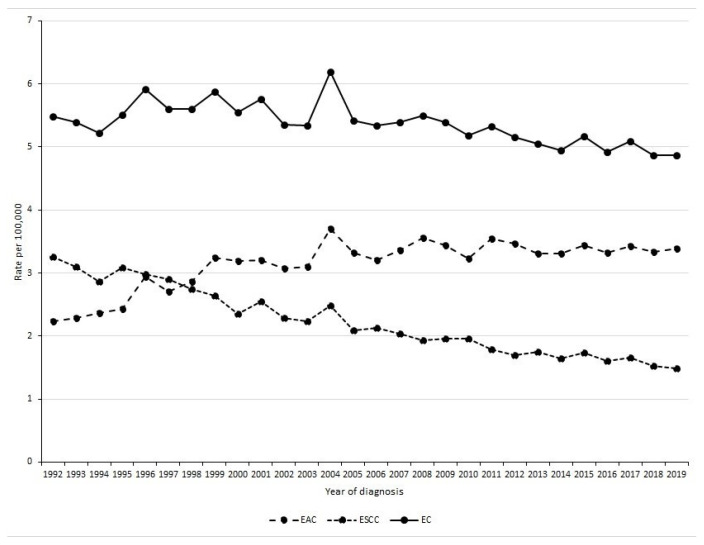
Time trends of age-standardized incidence rates for esophageal cancer (EC) overall, esophageal adenocarcinoma (EAC), and esophageal squamous cell carcinoma (ESCC).

Between 1992 and 2019, 38,025 persons were newly diagnosed with overall EC in the SEER 12 registries. The frequency of new EC cases increased from 1065 in 1992 to 1673 in 2019, an increase of 57.1% (Supplementary Table S1). The age-adjusted rate for the entire period was 5.32 per 100,000 (95% CI 5.27, 5.38), decreasing from 5.49 per 100,000 in 1992 to 4.87 per 100,000 in 2019 (Figure 1). Joinpoint regression did not identify significant inflection points such that EC rates decreased linearly at a rate of 0.54% per year (95% CI −0.75%, −0.33%) between 1992 to 2019 (Table 1). Table 2 shows that for EAC, rates increased by 5.17% per year (95% CI 3.28%, 7.10%) from 1992 to 2000, and were stable between 2000 and 2019 (APC, 0.22%; 95% CI −0.16%, 0.60%). The AAPC for the period 1992 to 2019 was 1.66% (95%CI: 1.09%, 2.24%). For ESCC, there was a significant linear decline in incidence rate from 1992 to 2019 (Table 3) with an AAPC of −2.85% (95% CI −3.05%, −2.65%). Figure 1 shows trends in EAC and ESCC rates over time.

### 3.2. Age Group

Age-specific incidence rates over time are shown in Supplementary Tables S2–S4. The highest age-specific incidence rates for overall EC were observed among persons aged 80–84 (22.99 per 100,000), 75–79 (22.02 per 100,000), and 70–74 (19.84 per 100,000) years. Between 1992 and 2019, age-group-specific AAPCs for overall EC were negative (indicating decreasing rates) for persons aged 45–49 (APC = −1.54%; 95% CI, −2.34%, −0.73%), 50–54 (APC = −1.28%; 95% CI, −1.82%, −0.74%), 55–59 (APC = −1.44%; 95% CI, −1.85%, −1.02%), 60–64 (APC = −0.85%: 95% CI, −1.25%, −0.45%), and 70–74 (APC = −0.60%; 95% CI, −0.95%, −0.24%) years (Table 1). For persons aged 65–69 and 75–79, there was an increase in age-specific incidence rates from 1992 to 1999 and 1992 to 2007, respectively, as indicated by an inflection point whereby rates decreased thereafter.

When examined by sub-type, the incidence rate of EAC increased in most but not all age groups, and the rate of change and linearity or not of trends varied (Table 2). For example, EAC rates remained stable for persons aged 45–49 years between 1992 and 2019 (APC = −0.13%; 95% CI: −1.24%, 0.99%), while EAC rates increased initially (1992–2000, APC = 5.23%; 95% CI: 0.71%, 9.95%) and then decreased by 1.03% per year (95% CI: −1.90%, −0.16%) from 2000 to 2019 for persons aged 55–59 years. For the 65–69 age group, EAC rates increased at a rate of 8.26% (95% CI: 4.19%, 12.48%) annually from 1992 to 1999 and then were stable through 2019. For ESCC, a consistent declining trend was observed for all the age groups from 1992 to 2019 (Table 3).

### 3.3. Sex

Incidence rates over time by sex are shown in Supplementary Table S5. Overall, the incidence rate of EC in females declined linearly throughout the study period (APC −1.13%, 95% CI −1.40%, −0.85%). Rates were stable among males from 1992 to 2004, followed by a significant decline from 2004 to 2019 (APC, −1.09%, 95% CI −1.49%, −0.68%). A parallelism test indicated that secular trends in EC rates were statistically significantly different between males and females (*p* < 0.001).

For EAC, rates increased from 1992 to 1999 in females (APC 7.10%, 95% CI 2.21%, 12.22%) and from 1992 to 2004 in males (APC 3.18%, 95% CI 2.21%, 4.15%) but were stable thereafter for both females and males. For ESCC, there was a marked linear decline in rates between 1992 and 2019 for both sexes as indicated by AAPCs of −2.46% (95% CI, −2.82%, −2.09%) in females and −3.17% (95% CI, −3.39%, −2.95%) in males.

### 3.4. Race/Ethnicity

Incidence rates over time by race/ethnicity are shown in Supplementary Tables S6–S8. EC rates decreased by 0.49% annually among NHWs from 2004 to 2019 (95%CI: −0.91%, −0.06%; however, the AAPC for NHWs indicated an overall increasing burden over the study period (AAPC, 0.43%; 95% CI: 0.07%, 0.80%) (Table 1). There was no inflection point identified, and thus the AAPC for overall EC indicates linear declines in EC rates for NHBs (AAPC, −4.41%; 95% CI, −4.75%, −4.07%), Hispanics (AAPC, −0.72%; 95% CI, −1.23%, −0.21%), and APIs (AAPC, −1.44%; 95% CI, −2.06%, −0.81%).

The race/ethnic-specific incidence trends were different for EAC versus ESCC. While EAC rates increased among NHWs and Hispanics at a rate of 2.21% (95%CI: 1.67%, 2.76%) and 0.95% (95%CI: 0.17%, 1.74%) per year, respectively (Table 2), ESCC rates among all race/ethnic groups showed significant negative linear trends (Table 3). There is however evidence that the rate of change in EAC incidence among NHWs slowed in 2000–2019 relative to an increase of 6.13% per year in 1992–2000 (95%CI: 4.36%, 7.93%). The highest declining trend for ESCC was observed in NHBs (APC = −5.41%; 95%CI: −5.79, −5.04). For NHBs and APIs, we had too few cases to examine EAC trends (only 2.29% of cases were NHB and 3.10% were APIs, Table 2).

### 3.5. Geography

Geographic locations except Atlanta demonstrated a declining trend in overall EC incidence rates (Table 4). There was an inflection point for Connecticut (AAPC −1.63%; 95% CI −2.42%, −0.82%) in 2016, where the APC from 1992 to 2016 declined by 0.97% (95% CI, −1.26, −0.68) while in 2016–2019, it declined by 6.71% without reaching statistical significance. There was also an inflection point for Los Angeles (AAPC −1.32%; 95% CI −1.70%, −0.95%) in 2013, where the APC from 1992 to 2013 was −0.90% (95% CI −1.14%, −0.65%) and the APC from 2013 to 2019 was −2.79% (95% CI −4.32%, −1.24%).

### 3.6. Age-Period-Cohort Models

**Figure 2:**
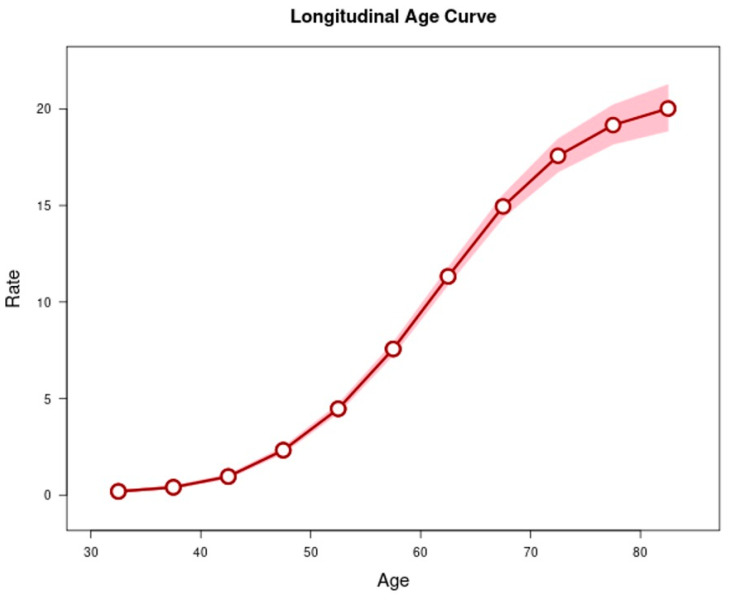
Longitudinal age curves of esophageal cancer in SEER 12 from 1992 to 2019 and corresponding 95% confidence intervals.

**Figure 3:**
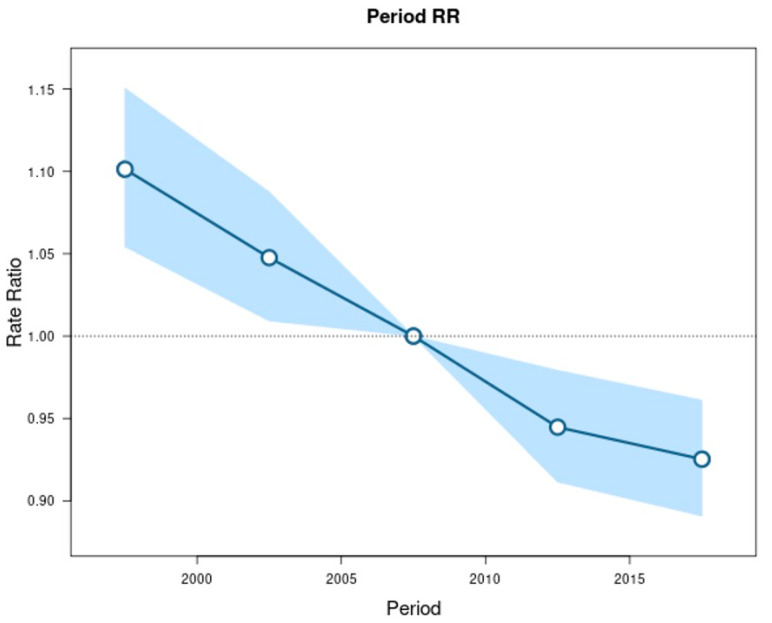
Incidence rate ratios by period (reference cohort 2005–2009) for esophageal cancer incidence in SEER 12. Shaded bands indicate the 95% confidence interval.

**Figure 4:**
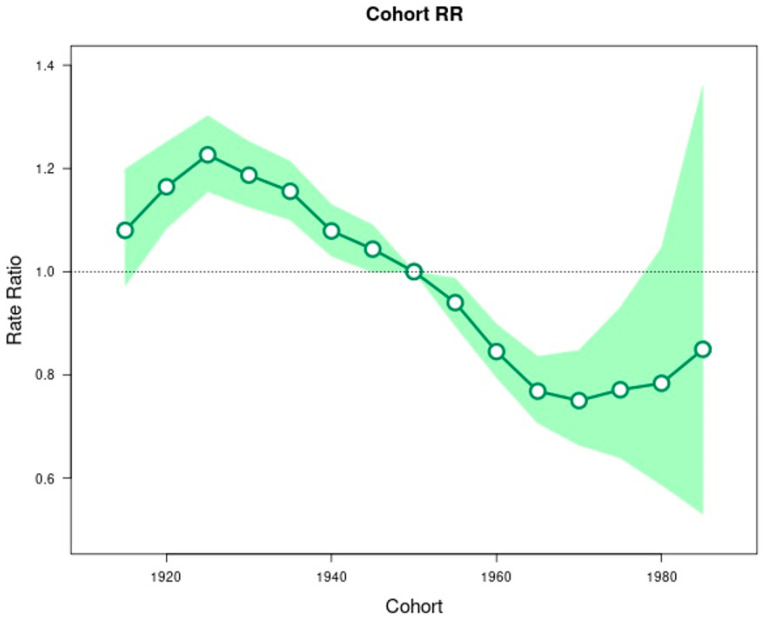
Incidence rate ratios by birth cohort (reference = cohort 1950) for esophageal cancer incidence in SEER 12 database. Shaded bands indicate the 95% confidence interval.

The longitudinal age curve shows that the rate of overall EC increases with increasing age (Figure 2). In addition to the age effect, there was evidence of a period effect (Figure 3) and birth cohort effect (Figure 4) on EC trends. Looking at the subtypes separately, such as EC overall, there were strong period and birth cohort effects for ESCC such that rates decreased across successive calendar periods (Supplementary Figure S1) and birth cohorts (Supplementary Figure S2). Relative to ESCC cohorts born circa 1950, the rates were 81% lower in cohorts born circa 1985 (rate ratio, 0.19; 95%CI: 0.04, 0.96). For EAC, rates were stable among the most recent calendar periods (Supplementary Figure S3) and successive birth cohorts since 1950 (Supplementary Figure S4).

## 4. Discussion

In our population-based study, we found that while the overall rate of EC has decreased since 1992, there are notable differences between secular trends for EAC and ESCC. The rates of EAC increased by over 5% annually between 1992 and 2000 before reaching a peak and stabilizing without an overall decline. On the other hand, ESCC rates continue to decline steadily in all age and race/ethnic groups and for both men and women.

Notably, while more recent studies on EC conclude that the incidence rate of EAC continues to rise, especially among younger adults [3], we used the most updated U.S. data and found that EAC incidence rates have peaked and are no longer increasing, regardless of age group. A particularly important finding from our study is that among individuals aged 45–49 years, EAC rates remained stable (with an AAPC of −0.13) over the course of the study period (1992–2019) and decreased in persons aged 50–54 (APC, −1.15%) and 55–59 (APC, −1.03%) years from 2000 to 2019. The additional years of data used in our analysis, especially for sub-group analyses, helped to more reliably ascertain whether EAC rates have peaked.

Given the rise of obesity and gastroesophageal reflux disease, which are the major risk factors for the development of Barrett’s esophagus [16,17], the precursor lesion to EAC, it is not clear why the rising rate of both Barrett’s esophagus and EAC has slowed in the 21st century. We postulate that protective factors including acid suppression therapy [18], statins [19,20], stabilization of obesity rates in some subsets of the U.S. population [21], and increased awareness and surveillance of Barrett’s esophagus may have contributed to tempering the rise in EAC. In addition, our age-period-cohort analysis highlighted a cohort effect, demonstrating a stabilization of EAC for younger cohorts born after 1950. This, along with the overall stabilization of rates since 2000, suggests that EAC rates have slowed down in the U.S. It is, however, also important to note that although the rates of EAC among NHWs have shown a 90% decline in the rate of increase in 2000–2019 relative to 1992–2000, it has yet to show a significant negative trend. Without a definitive cause of this decline, it is evident that additional study needs to be undertaken.

The rates of ESCC declined linearly from 1992 and continue to decrease overall across all age groups, sex, and race/ethnicity. The largest declines in ESCC rates were seen in the 55–59 age group, in males, and among NHBs. Our data build upon prior publications demonstrating a marked decrease in the incidence rate of ESCC in the Western world over the last several decades [5,22]. Given the strong correlation between known risk factors and the development of ESCC (alcohol consumption and cigarette smoking: individually confer a threefold to fivefold increased risk of ESCC development, while together they confer a twenty-fold increased risk of ESCC development in smokers who drink heavily), the decreased rates of smoking and alcohol consumption likely explain the decreasing incidence of ESCC [23]. Our age-period-cohort analysis revealed a period effect and an even stronger cohort effect, with earlier periods and older birth cohorts experiencing higher rates of ESCC compared to later periods and younger birth cohorts, respectively. This is congruent with prior studies [5] and suggests that a strong period and cohort effect remains such that ESCC rates continue to decline in the U.S.

The strengths of our study include results being supported by population-based data with a low likelihood of information bias, as these data were collected prospectively and independently of our analyses. A limitation of our study is that it was based on the SEER 12 cancer registry data and that no information on individual EAC/ESCC risk factors was available. As such, our study is unable to provide any direct evidence about the role of specific exposures or intervention effects we noted for the EAC/ESCC incidence trends. While we were able to determine that incidence rates for overall EC were decreasing in younger patients, the data were also limited in the number of younger patients in the registry, and we were unable to perform joinpoint analysis on patients aged <45 years for EAC or <50 years for ESCC.

## 5. Conclusions

We found that ESCC rates have been steadily declining, while EAC rates rose rapidly before stabilizing in 2000. The trend of decreasing incidence of ESCC was observed almost uniformly across subgroups of age, sex, and race/ethnicity, while trends among these subgroups were more variable for EAC (e.g., stable in those aged 45–49 years, increasing and then declining in those aged 50–59 years). The EAC incidence trend among NHWs has also shown signs of slowing between 2000 and 2019 while still maintaining a positive linear trend overall (1992–2019). A cohort effect for EAC was observed among people born during 1950, but EAC rates were stable across successive generations born between 1950 and 1985. Given the continued rising rates of known EAC risk factors, including obesity and gastroesophageal reflux disease, there is a need to continue monitoring trends for changes in incidence rates.

## Figures and Tables

**Table 1:** Annual percent change (APC) and average annual percent change (AAPC) in EC rates over time, overall, and by age group, sex, and race/ethnicity.

Characteristics	Joinpoint Segment Year Start	Joinpoint Segment Year End	APC (95% CI)	*p*-Value	AAPC (95% CI)	*p*-Value
Overall	1992	2019	−0.54 (−0.75, −0.33)	<0.001		
Age, years						
45–49	1992	2019	−1.54 (−2.34, −0.73)	<0.001		
50–54	1992	2019	−1.28 (−1.82, −0.74)	<0.001		
55–59	1992	2019	−1.44 (−1.85, −1.02)	<0.001		
60–64	1992	2019	−0.85 (−1.25, −0.45)	<0.001		
65–69	1992	1999	1.77 (−0.20, 3.77)	0.076	−0.42 (−0.96, 0.12)	0.125
	1999	2019	−1.18 (−1.53, −0.82)	<0.001		
70–74	1992	2019	−0.60 (−0.95, −0.24)	0.002		
75–79	1992	2007	1.07 (0.33, 1.82)	0.006	−0.03 (−0.59, 0.53)	0.908
	2007	2019	−1.40 (−2.33, −0.45)	0.006		
80–84	1992	2019	0.46 (−0.24, 1.17)	0.187		
85+	1992	2019	0.07 (−0.63, 0.77)	0.845		
Sex						
Female	1992	2019	−1.13 (−1.40, −0.85)	<0.001		
Male	1992	2004	0.46 (−0.20, 1.12)	0.162	−0.40 (−0.75, −0.05)	0.025
	2004	2019	−1.09 (−1.49, −0.68)	<0.001		
Race/Ethnicity						
NHW	1992	2004	1.59 (0.91, 2.28)	<0.001	0.43 (0.07, 0.80)	<0.001
	2004	2019	−0.49 (−0.91, −0.06)	0.028		
NHB	1992	2019	−4.41 (−4.75, −4.07)	<0.001		
Hispanic	1992	2019	−0.72 (−1.23, −0.21)	0.007		
API	1992	2019	−1.44 (−2.06, −0.81)	<0.001		

AAPC, average annual percent change; APC, annual percent change; API, Asians, and Pacific Islanders; CI, confidence interval; EC, esophageal carcinoma; NHB, non-Hispanic Black; NHW, non-Hispanic White.

**Table 2:** Annual percent change (APC) and average annual percent change (AAPC) in EAC rates over time, overall, and by age group, sex, and race/ethnicity.

Characteristics	Joinpoint Segment Year Start	Joinpoint Segment Year End	APC (95% CI)	*p*-Value	AAPC (95% CI)	*p*-Value
Overall	1992	2000	5.17 (3.28, 7.10)	<0.001	1.66 (1.09, 2.24)	<0.001
	2000	2019	0.22 (−0.16, 0.60)	0.245		
Age, years						
45–49	1992	2019	−0.13 (−1.24, 0.99)	0.808		
50–54	1992	2000	6.43 (1.32, 11.80)	0.015	1.04 (−0.50, 2.61)	0.188
	2000	2019	−1.15 (−2.15, −0.14)	0.027		
55–59	1992	2000	5.23 (0.71, 9.95)	0.025	0.78 (−0.58, 2.17)	0.264
	2000	2019	−1.03 (−1.90, −0.16)	0.022		
60–64	1992	2019	0.94 (0.37, 1.52)	0.002		
65–69	1992	1999	8.26 (4.19, 12.48)	<0.001	2.06 (1.02, 3.11)	<0.001
	1999	2019	−0.02 (−0.61, 0.56)	0.936		
70–74	1992	2000	4.43 (1.48, 7.46)	0.005	1.77 (0.85, 2.69)	<0.001
	2000	2019	0.67 (0.05, 1.28)	0.035		
75–79	1992	2004	3.51 (1.80, 5.24)	<0.001	1.60 (0.73, 2.49)	<0.001
	2004	2019	0.11 (−0.86, 1.09)	0.821		
80–84	1992	2019	1.89 (0.92, 2.88)	<0.001		
85+	1992	2019	1.26 (0.24, 2.28)	0.017		
Sex						
Female	1992	1999	7.10 (2.21, 12.22)	0.006	2.04 (0.77, 3.32)	0.002
	1999	2019	−0.32 (−0.38, 1.03)	0.357		
Male	1992	2004	3.18 (2.21, 4.15)	<0.001	1.19 (0.70, 1.68)	<0.001
	2004	2019	−0.37 (−0.89, 0.15)	0.15		
Race/Ethnicity						
NHW	1992	2000	6.13 (4.36, 7.93)	<0.001	2.21 (1.67, 2.76)	<0.001
	2000	2019	0.61 (0.24, 0.97)	0.002		
Hispanic	1992	2019	0.95 (0.17, 1.74)	0.018		

Abbreviations: AAPC, average annual percent change; APC, annual percent change; API, Asian and Pacific Islanders; CI, confidence interval; NHB, non-Hispanic Black; NHW, non-Hispanic White. API and NHB subgroups were excluded due to small numbers of cases per year.

**Table 3:** Annual percent change (APC) and average annual percent change (AAPC) in ESCC rates over time, overall, and by age group, sex, and race/ethnicity.

Characteristics	Joinpoint Segment Year Start	Joinpoint Segment Year End	APC (95% CI)	*p*-Value	AAPC (95% CI)	*p*-Value
Overall	1992	2019	−2.85 (−3.05, −2.65)	<0.001		
Age						
50–54	1992	2019	−3.48 (−4.31, −2.64)	<0.001		
55–59	1992	2002	−7.34 (−10.73, −3.82)	<0.001	−3.86 (−5.47, −2.23)	<0.001
	2002	2019	−1.76 (−3.48, 0.00)	0.050		
60–64	1992	2011	−4.66 (−5.56, −3.75)	<0.001	−2.95 (−4.08, −1.81)	<0.001
	2011	2019	1.23 (−2.25,4.84)	0.478		
65–69	1992	2019	−3.17 (−3.59, −2.76)	<0.001		
70–74	1992	2019	−3.13 (−3.70, −2.56)	<0.001		
75–79	1992	2008	−0.88 (−1.87, 0.13)	0.084	−2.00 (−2.90, −1.09)	<0.001
	2008	2019	−3.60 (−5.41, −1.76)	0.001		
80–84	1992	2019	−1.62 (−2.30, −0.92)	<0.001		
85+	1992	2019	−1.84 (−2.57, −1.10)	<0.001		
Sex						
Female	1992	2019	−2.46 (−2.82, −2.09)	<0.001		
Male	1992	2019	−3.17 (−3.39, −2.95)	<0.001		
Race/Ethnicity						
NHW	1992	2019	−2.44 (−2.71, −2.16)	<0.001		
NHB	1992	2019	−5.41 (−5.79, −5.04)	<0.001		
Hispanic	1992	2019	−2.83 (−3.48, −2.17)	<0.001		
API	1992	2019	−2.26 (−2.93, −1.57)	<0.001		

Abbreviations: AAPC, average annual percent change; APC, annual percent change; API, Asian and Pacific Islanders; CI, confidence interval; NHB, non-Hispanic Black; NHW, non-Hispanic White.

**Table 4:** Annual percent change (APC) and average annual percent change (AAPC) in EC rates over time by SEER 12 registry.

SEER 12 Registry	Joinpoint Segment Year Start	Joinpoint Segment Year End	APC (95% CI)	*p*-Value	AAPC (95% CI)	*p*-Value
Connecticut	1992	2016	−0.97 (−1.26, −0.68)	<0.001	−1.63 (−2.42, −0.82)	<0.001
	2016	2019	−6.71 (−13.33,0.41)	0.063		
Atlanta	1992	2019	−0.02 (−0.47, 0.43)	0.926		
San Francisco–Oakland	1992	2019	−1.38 (−1.61, −1.16)	<0.001		
San Jose–Monterey	1992	2019	−1.00 (−1.34, −0.66)	<0.001		
Hawaii	1992	2019	−3.49 (−3.76, −3.22)	<0.001		
Iowa	1992	2019	−1.23 (−1.55, −0.91)	<0.001		
Los Angeles	1992	2013	−0.90 (−1.14, −0.65)	<0.001	−1.32 (−1.70, −0.95)	<0.001
	2013	2019	−2.79 (−4.32, −1.24)	0.001		
New Mexico	1992	2019	−0.96 (−1.58, −0.35)	0.004		
Seattle–Puget Sound	1992	2019	−0.96 (−1.24, −0.68)	<0.001		
Utah	1992	2019	−1.11 (−1.59, −0.63)	<0.001		

Alaska and Rural Georgia were excluded from Registry stratified analyses due to too-suppressed cells for years except 2017 and 2019 for Alaska and 2018 for Rural Georgia.

## Data Availability

The data presented in this study are available on request from the corresponding author.

## Supplementary Materials

The following supporting information can be downloaded at: https://www.mdpi.com/article/10.3390/cancers14246049/s1, Figure S1: Incidence rate ratios by period (reference cohort 2005–2009) for esophageal squamous cell carcinoma incidence in SEER 12. Shaded bands indicate the 95% confidence interval. Figure S2. Incidence rate ratios by birth cohort (reference = cohort 1955) for esophageal squamous cell carcinoma incidence in SEER 12 database. Shaded bands indicate the 95% confidence interval. Figure S3. Incidence rate ratios by period (reference cohort 2005–2009) for esophageal adenocarcinoma incidence in SEER 12. Shaded bands indicate the 95% confidence interval. Figure S4. Incidence rate ratios by birth cohort (reference = cohort 1955) for esophageal adenocarcinoma incidence in SEER 12 database. Shaded bands indicate the 95% confidence interval. Table S1. Annual frequencies and age-adjusted incidence rates of EC, EAC, and ESCC: SEER 12 registries between 1992 and 2019. Table S2. Age-adjusted incidence rates of EC by age group: SEER 12 registries between 1992 and 2019. Table S3. Age-adjusted incidence rates of EAC by age group: SEER 12 registries between 1992 and 2019. Table S4. Age-adjusted incidence rates of ESCC by age group: SEER 12 registries between 1992 and 2019. Table S5. Age-adjusted incidence rates of EC, EAC, and ESCC by sex: SEER 12 registries between 1992 and 2019. Table S6. Age-adjusted incidence rates of EC by race/ethnicity: SEER 12 registries between 1992 and 2019. Table S7. Age-adjusted incidence rates of EAC by race/ethnicity: SEER 12 registries between 1992 and 2019. Table S8. Age-adjusted incidence rates of ESCC by race/ethnicity: SEER 12 registries between 1992 and 2019.
