# Supplementary material for: Trends in Esophageal Adenocarcinoma and Esophageal Squamous Cell Carcinoma Incidence in the United States from 1992 to 2019
Source: Cancers (Basel). 2022 Dec 8;14(24):6049. doi: 10.3390/cancers14246049 (PMC9775957; doi:10.3390/cancers14246049)
Supplement: Supplementary file 1 [file cancers-14-06049-s001.zip › cancers-1991419-supplementary.pdf]

cancers

MDPI

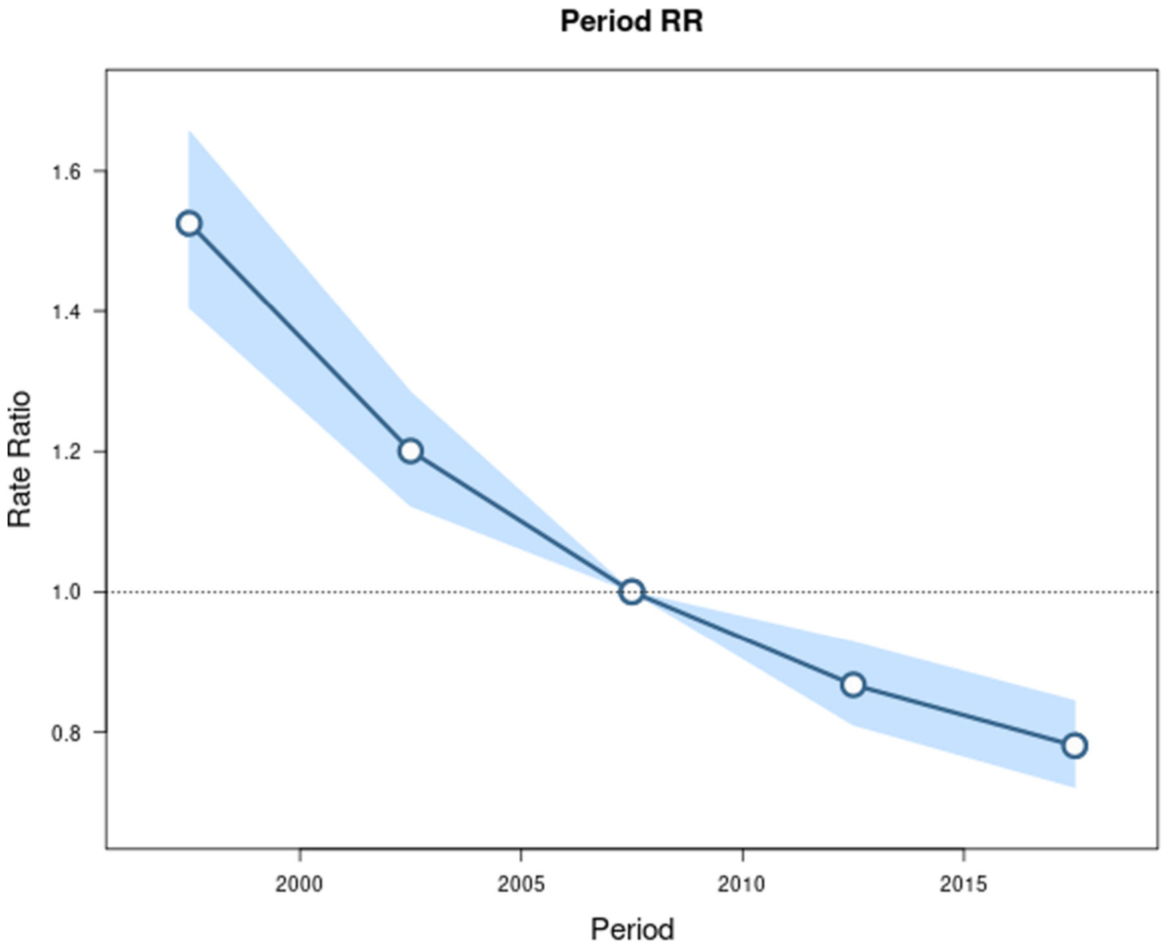

Supplemental Figure S1. Incidence rate ratios by period (reference cohort 2005-2009) for esophageal squamous cell carcinoma incidence in SEER 12. Shaded bands indicate the 95% confidence interval.

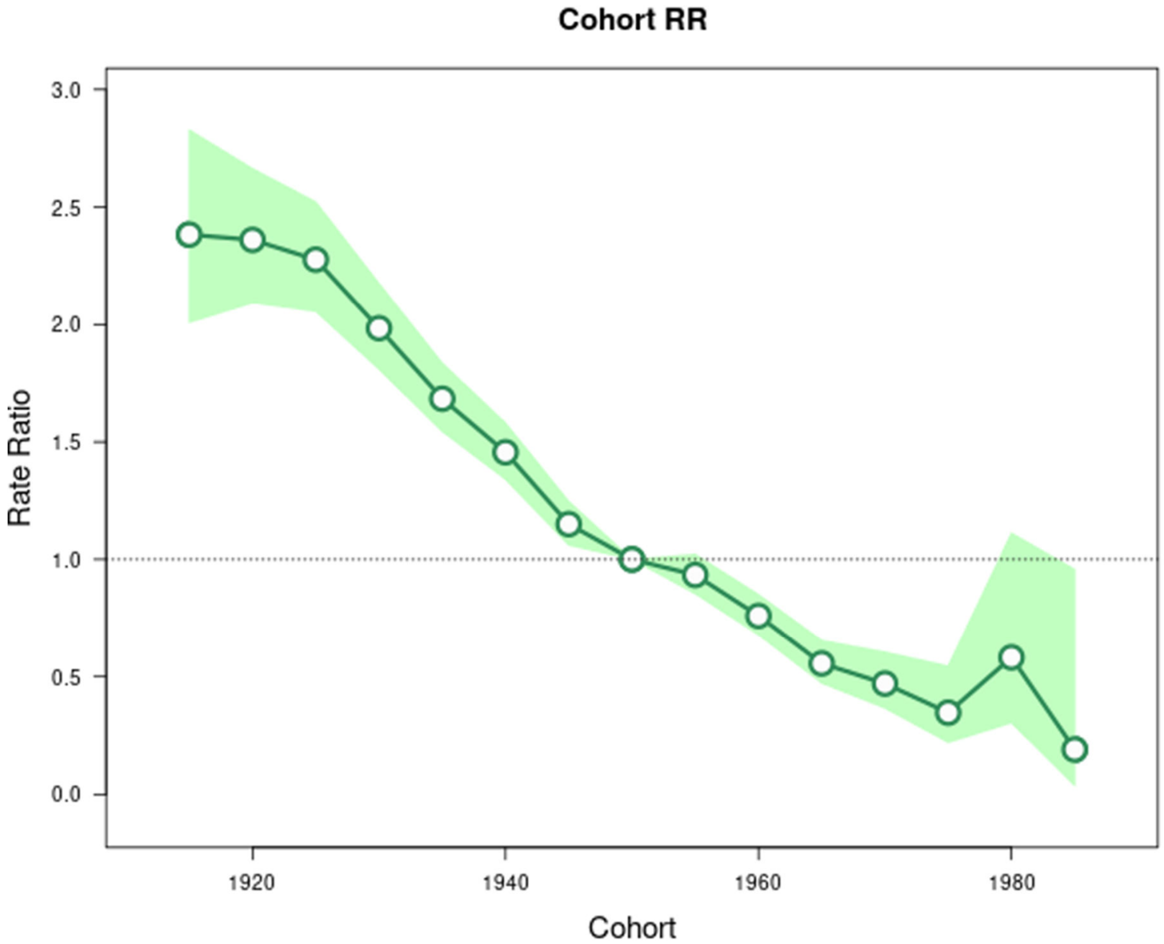

Supplemental Figure S2. Incidence rate ratios by birth cohort (reference = cohort 1955) for esophageal squamous cell carcinoma incidence in SEER 12 database. Shaded bands indicate the 95% confidence interval.

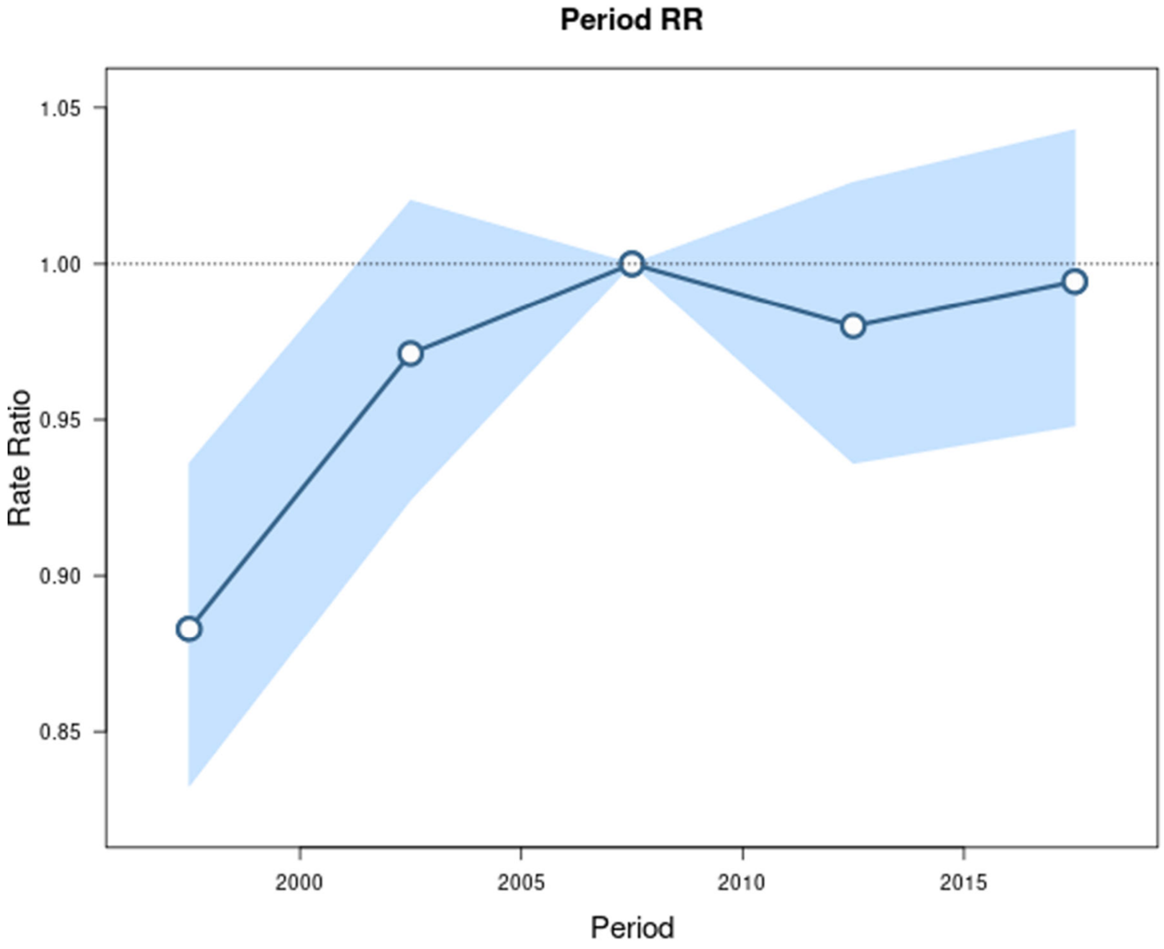

Supplemental Figure S3. Incidence rate ratios by period (reference cohort 2005-2009) for esophageal adenocarcinoma incidence in SEER 12. Shaded bands indicate the 95% confidence interval.

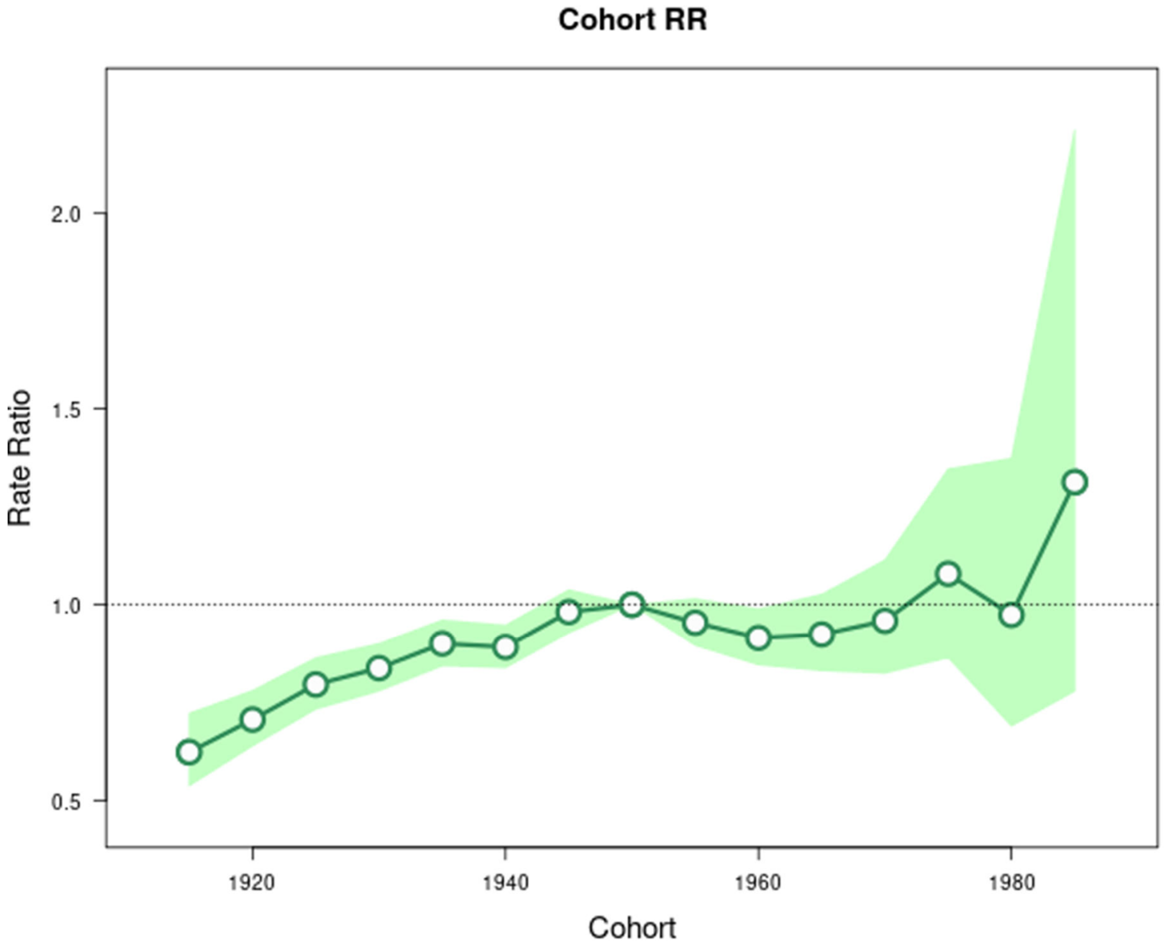

Supplemental Figure S4. Incidence rate ratios by birth cohort (reference = cohort 1955) for esophageal adenocarcinoma incidence in SEER 12 database. Shaded bands indicate the 95% confidence interval.

Supplementary Table S1. Annual frequencies and age-adjusted incidence rates of EC, EAC and ESCC: SEER 12 registries between 1992 and 2019.

Year	Incident EC	Age-adjusted rate per 100,000 (95% CI)	Incident EAC	Age-adjusted rate per 100,000 (95% CI)	Incident ESCC	Age-adju[illegible] per 100,00[illegible]
Total	38,025	5.32 (5.27-5.38)	22,798	3.19 (3.14-3.23)	15,227	2.14 (2.[illegible]
1992	1,065	5.49 (5.16-5.83)	431	2.23 (2.02-2.45)	634	3.25 (3.[illegible]
1993	1,066	5.39 (5.07-5.72)	452	2.29 (2.08-2.51)	614	3.10 (2.[illegible]
1994	1,045	5.23 (4.91-5.55)	474	2.37 (2.16-2.59)	571	2.86 (2.[illegible]
1995	1,118	5.51 (5.19-5.85)	494	2.43 (2.22-2.66)	624	3.08 (2.[illegible]
1996	1,217	5.92 (5.59-6.26)	607	2.94 (2.71-3.19)	610	2.97 (2.[illegible]
1997	1,175	5.60 (5.29-5.93)	568	2.70 (2.48-2.93)	607	2.90 (2.[illegible]
1998	1,194	5.60 (5.29-5.93)	610	2.86 (2.64-3.09)	584	2.74 (2.[illegible]
1999	1,276	5.87 (5.56-6.21)	706	3.24 (3.01-3.49)	570	2.63 (2.[illegible]
2000	1,231	5.54 (5.24-5.86)	710	3.19 (2.96-3.43)	521	2.36 (2.[illegible]

2001	1,292	5.75 (5.44-6.08)	720	3.20 (2.97-3.44)	572	2.55 (2.[illegible]
2002	1,229	5.36 (5.06-5.67)	707	3.07 (2.85-3.31)	522	2.29 (2.[illegible]
2003	1,246	5.34 (5.05-5.65)	727	3.10 (2.88-3.34)	519	2.24 (2.[illegible]
2004	1,476	6.19 (5.88-6.52)	889	3.70 (3.46-3.96)	587	2.49 (2.[illegible]
2005	1,314	5.41 (5.12-5.71)	809	3.32 (3.09-3.55)	505	2.10 (1.[illegible]
2006	1,323	5.34 (5.05-5.64)	802	3.21 (2.99-3.44)	521	2.13 (1.[illegible]
2007	1,363	5.39 (5.10-5.68)	850	3.35 (3.13-3.59)	513	2.03 (1.[illegible]
2008	1,426	5.49 (5.20-5.79)	932	3.56 (3.33-3.80)	494	1.93 (1.[illegible]
2009	1,445	5.39 (5.12-5.68)	928	3.44 (3.22-3.67)	517	1.96 (1.[illegible]
2010	1,419	5.18 (4.91-5.46)	888	3.22 (3.01-3.45)	531	1.96 (1.[illegible]
2011	1,501	5.32 (5.05-5.60)	1,003	3.54 (3.32-3.77)	498	1.78 (1.[illegible]
2012	1,478	5.16 (4.90-5.44)	996	3.46 (3.25-3.69)	482	1.70 (1.[illegible]
2013	1,504	5.05 (4.80-5.32)	982	3.31 (3.10-3.53)	522	1.74 (1.[illegible]
2014	1,495	4.94 (4.69-5.21)	998	3.31 (3.10-3.52)	497	1.64 (1.[illegible]
2015	1,609	5.17 (4.91-5.43)	1,077	3.44 (3.23-3.65)	532	1.73 (1.[illegible]
2016	1,564	4.92 (4.68-5.18)	1,060	3.32 (3.12-3.53)	504	1.60 (1.[illegible]
2017	1,659	5.08 (4.83-5.34)	1,109	3.43 (3.23-3.64)	550	1.65 (1.[illegible]
2018	1,622	4.87 (4.63-5.12)	1,108	3.34 (3.14-3.54)	514	1.53 (1.[illegible]
2019	1,673	4.87 (4.63-5.11)	1,161	3.39 (3.19-3.59)	512	1.48 (1.[illegible]

CI, confidence interval; EC, esophageal cancer

Supplementary Table S2. Age-adjusted incidence rates of EC by age group: SEER 12 registries between 1992 and 2019.

Year	45-49	50-54	55-59	60-64	65-69	70-74	75-79	80-84	85+
1992	2.77	4.96	8.73	13.96	17.33	18.78	19.95	17.64	18.64
1993	2.17	3.65	10.75	14.47	15.84	20.58	20.45	17.52	11.61
1994	1.65	4.33	7.35	14.26	14.93	20.53	19.93	22.56	14.32
1995	2.41	4.90	7.71	12.53	17.78	20.01	23.22	17.94	18.98
1996	2.07	5.07	10.01	13.38	17.15	23.10	21.04	22.88	19.82
1997	2.45	4.25	8.46	14.10	17.18	23.29	20.16	19.66	17.12
1998	2.34	3.29	9.13	11.84	17.36	19.00	23.25	25.84	20.40
1999	2.23	4.88	8.23	13.96	19.82	24.16	21.82	22.01	16.68
2000	2.54	4.83	8.99	10.59	17.30	18.81	22.74	24.04	16.42
2001	2.13	4.89	7.03	12.41	17.84	22.84	23.58	23.19	21.25
2002	2.39	4.47	7.38	12.05	16.40	20.65	20.89	20.00	19.82
2003	2.08	4.39	7.05	10.18	16.91	21.25	21.52	21.19	20.85
2004	1.98	4.97	8.47	13.20	18.36	21.51	25.32	31.09	25.60
2005	1.81	4.53	7.90	10.41	16.76	20.16	23.61	24.17	18.14
2006	1.76	4.51	7.31	12.32	16.79	20.43	22.09	23.95	15.85
2007	2.37	3.88	7.54	10.85	17.58	18.38	24.38	24.36	16.63
2008	2.65	3.45	8.00	10.84	16.11	19.44	23.40	27.82	20.86
2009	1.73	4.35	7.04	13.38	14.71	19.63	22.54	24.50	20.14
2010	2.22	3.83	6.85	11.36	15.83	19.11	23.22	18.07	20.85
2011	1.66	4.06	7.31	12.25	16.08	19.02	22.11	25.73	19.33
2012	1.76	3.59	6.55	10.45	15.81	18.79	24.47	21.28	19.95
2013	1.91	3.40	6.83	11.85	15.85	19.84	21.56	19.55	17.49
2014	1.35	3.54	6.51	10.76	14.74	15.50	21.48	27.88	16.52
2015	2.22	3.47	6.38	11.21	15.19	19.56	21.35	23.31	21.15

2016	1.18	3.60	6.14	10.06	15.05	18.48	22.15	23.66	18.35
2017	1.67	3.17	7.41	11.32	14.70	18.88	22.33	24.20	16.42
2018	1.53	3.74	6.18	10.73	13.57	19.65	20.10	24.75	15.85
2019	1.48	3.55	6.16	11.78	14.91	18.12	18.64	20.42	19.64

Supplementary Table S3. Age-adjusted incidence rates of EAC by age group: SEER 12 registries between 1992 and 2019.

Year	45-49	50-54	55-59	60-64	65-69	70-74	75-79	80-84	85+
1992	1.10	2.07	3.13	5.51	6.40	7.14	9.68	7.92	7.95
1993	1.46	1.43	4.28	5.97	5.56	9.27	9.50	7.01	5.54
1994	0.77	1.82	3.56	6.06	6.16	9.04	9.61	12.77	4.09
1995	1.21	2.30	3.27	6.00	7.77	8.11	8.99	8.04	9.62
1996	1.37	2.65	5.00	5.75	9.09	10.49	10.45	10.64	10.74
1997	1.40	2.20	4.45	6.47	8.26	9.83	8.71	9.83	8.79
1998	1.11	2.31	4.26	5.70	8.49	9.60	12.01	14.27	9.19
1999	1.49	2.83	4.87	8.50	11.13	11.28	11.04	11.10	10.83
2000	1.69	2.84	5.51	5.46	9.19	11.37	13.10	14.13	7.58
2001	0.98	2.89	4.40	6.85	9.75	11.74	13.46	12.21	12.58
2002	1.43	2.83	4.85	6.53	9.46	11.62	11.62	10.26	11.33
2003	1.14	2.56	4.47	5.97	10.30	11.17	11.50	12.85	13.18
2004	1.24	3.48	5.55	7.91	10.89	10.92	14.84	17.90	16.36
2005	1.26	2.66	5.61	6.26	9.55	12.31	14.12	13.93	11.66
2006	1.21	2.76	5.21	8.00	9.76	12.17	11.92	12.29	9.44
2007	1.46	2.54	4.49	6.87	10.99	11.61	14.02	15.76	10.29
2008	2.00	2.60	5.70	6.88	10.13	12.92	12.84	18.60	12.58
2009	1.41	3.17	4.53	8.76	9.55	11.65	12.86	14.47	12.79
2010	1.35	2.18	4.28	7.66	10.75	11.65	13.71	10.15	12.82
2011	1.29	2.72	4.34	9.11	10.70	11.10	14.19	17.83	12.74
2012	1.24	2.30	4.72	7.48	9.90	12.12	15.70	14.03	14.12
2013	1.30	2.03	4.00	7.71	10.40	13.37	13.85	12.46	12.52
2014	1.04	2.22	4.02	6.96	10.78	10.09	14.01	19.37	10.28
2015	1.72	2.31	4.55	7.46	10.76	13.41	12.61	14.08	13.56
2016	0.64	2.65	4.13	7.28	10.44	12.55	14.33	15.15	11.30
2017	1.29	2.32	4.82	7.34	9.01	12.78	14.92	16.89	10.90
2018	1.19	2.64	4.28	7.19	8.96	13.62	13.20	17.78	10.13
2019	0.97	2.51	4.42	7.77	10.45	12.49	12.81	14.38	13.60

Supplementary Table S4. Age-adjusted incidence rates of ESCC by age group: SEER 12 registries between 1992 and 2019.

Year	50-54	55-59	60-64	65-69	70-74	75-79	80-84	85+
1992	2.89	5.60	8.44	10.94	11.64	10.27	9.73	10.69
1993	2.22	6.46	8.51	10.28	11.31	10.94	10.51	6.07
1994	2.51	3.79	8.20	8.77	11.49	10.32	9.79	10.23
1995	2.60	4.44	6.53	10.02	11.90	14.24	9.90	9.37

1996	2.41	5.00	7.63	8.06	12.61	10.59	12.24	9.07
1997	2.04	4.01	7.63	8.92	13.46	11.44	9.83	8.33
1998	0.98	4.88	6.13	8.88	9.39	11.24	11.57	11.21
1999	2.05	3.36	5.47	8.69	12.88	10.79	10.91	5.85
2000	2.00	3.48	5.13	8.11	7.44	9.64	9.91	8.84
2001	2.00	2.63	5.56	8.09	11.10	10.12	10.97	8.66
2002	1.64	2.54	5.52	6.95	9.04	9.27	9.75	8.49
2003	1.82	2.58	4.20	6.61	10.08	10.02	8.34	7.67
2004	1.49	2.93	5.30	7.47	10.59	10.48	13.18	9.24
2005	1.87	2.29	4.15	7.21	7.85	9.49	10.24	6.48
2006	1.75	2.10	4.33	7.03	8.26	10.17	11.65	6.41
2007	1.35	3.05	3.98	6.59	6.77	10.36	8.60	6.34
2008	0.85	2.31	3.96	5.98	6.51	10.55	9.22	8.28
2009	1.18	2.51	4.62	5.16	7.97	9.68	10.02	7.35
2010	1.65	2.57	3.70	5.08	7.47	9.52	7.93	8.03
2011	1.34	2.96	3.14	5.38	7.92	7.91	7.89	6.59
2012	1.30	1.83	2.96	5.91	6.66	8.78	7.25	5.82
2013	1.37	2.84	4.14	5.45	6.47	7.71	7.10	4.98
2014	1.33	2.49	3.80	3.96	5.42	7.47	8.50	6.25
2015	1.16	1.83	3.75	4.44	6.15	8.75	9.23	7.59
2016	0.95	2.01	2.78	4.61	5.93	7.82	8.50	7.05
2017	0.85	2.59	3.97	5.69	6.11	7.41	7.31	5.52
2018	1.10	1.89	3.53	4.61	6.03	6.90	6.97	5.72
2019	1.04	1.74	4.01	4.46	5.63	5.83	6.04	6.03

Supplementary Table S5. Age-adjusted incidence rates of EC, EAC, and ESCC by sex: SEER 12 registries between 1992 and 2019.

EC			EAC			ESCC		
Year	Males	Females	Year	Males	Females	Year	Males	Females
1992	9.28	2.52	1992	4.44	0.49	1992	4.84	2.04
1993	9.18	2.40	1993	4.47	0.59	1993	4.71	1.81
1994	8.90	2.39	1994	4.64	0.61	1994	4.26	1.78
1995	9.07	2.70	1995	4.84	0.58	1995	4.24	2.12
1996	9.96	2.73	1996	5.62	0.84	1996	4.34	1.89
1997	9.32	2.65	1997	5.17	0.74	1997	4.14	1.91
1998	9.64	2.49	1998	5.67	0.69	1998	3.96	1.80
1999	10.08	2.53	1999	6.18	0.89	1999	3.90	1.64
2000	9.26	2.54	2000	6.06	0.89	2000	3.21	1.66
2001	10.09	2.33	2001	6.24	0.80	2001	3.84	1.53
2002	9.15	2.35	2002	5.90	0.83	2002	3.25	1.52
2003	9.32	2.18	2003	5.97	0.82	2003	3.35	1.36
2004	10.53	2.74	2004	7.29	0.87	2004	3.24	1.87
2005	9.43	2.19	2005	6.29	0.93	2005	3.14	1.26
2006	8.97	2.37	2006	5.98	0.94	2006	2.99	1.43

2007	9.30	2.24	2007	6.43	0.89	2007	2.86	1.35
2008	9.56	2.19	2008	6.80	0.95	2008	2.76	1.23
2009	9.30	2.21	2009	6.57	0.87	2009	2.73	1.34
2010	9.11	1.99	2010	6.29	0.72	2010	2.82	1.27
2011	9.25	2.11	2011	6.69	0.96	2011	2.56	1.15
2012	8.94	2.05	2012	6.67	0.82	2012	2.26	1.23
2013	8.59	2.10	2013	6.22	0.89	2013	2.37	1.21
2014	8.42	2.05	2014	6.16	0.94	2014	2.26	1.12
2015	8.83	2.11	2015	6.43	0.94	2015	2.41	1.17
2016	8.41	2.02	2016	6.25	0.88	2016	2.16	1.14
2017	8.92	1.87	2017	6.64	0.74	2017	2.28	1.13
2018	8.19	2.04	2018	6.20	0.92	2018	1.99	1.12
2019	8.26	2.02	2019	6.23	0.99	2019	2.03	1.02

Supplementary Table S6. Age-adjusted incidence rates of EC by race/ethnicity: SEER 12 registries between 1992 and 2019.

Year	NHW	NHB	API	Hispanic
1992	5.27	12.72	3.04	4.29
1993	5.12	11.21	4.19	4.15
1994	4.98	11.61	3.85	3.98
1995	5.50	10.82	3.55	3.89
1996	5.82	11.91	4.48	3.87
1997	5.68	9.78	4.10	3.88
1998	5.77	8.60	4.23	3.37
1999	6.19	8.60	3.80	4.75
2000	5.89	7.83	3.67	3.90
2001	6.24	7.19	3.45	4.05
2002	5.80	7.61	3.55	3.48
2003	5.93	8.03	2.70	3.03
2004	6.77	6.85	4.23	4.43
2005	5.97	6.25	3.37	3.99
2006	6.03	6.32	3.52	2.77
2007	6.21	6.00	2.80	3.48
2008	6.31	5.63	3.44	3.12
2009	6.21	6.21	2.61	3.58
2010	5.81	5.67	3.42	3.64
2011	6.32	4.81	2.61	3.69
2012	6.03	4.45	2.64	4.01
2013	5.97	5.13	2.60	3.28
2014	5.77	4.70	2.77	3.48
2015	6.10	3.81	3.00	3.80
2016	5.64	4.56	3.25	3.54
2017	6.09	4.14	3.22	3.31

2018	5.99	4.07	2.35	3.00
2019	5.88	3.48	3.03	3.51

Supplementary Table S7. Age-adjusted incidence rates of EAC by race/ethnicity: SEER 12 registries between 1992 and 2019.

Year	NHW	Hispanic
1992	2.67	1.23
1993	2.74	1.45
1994	2.76	1.96
1995	3.00	1.46
1996	3.55	2.03
1997	3.33	2.00
1998	3.60	1.87
1999	4.06	2.26
2000	4.03	1.90
2001	4.00	2.17
2002	3.97	1.86
2003	4.08	1.49
2004	4.69	2.44
2005	4.18	2.40
2006	4.26	1.45
2007	4.47	1.76
2008	4.69	1.80
2009	4.50	2.12
2010	4.27	1.77
2011	4.71	2.40
2012	4.48	2.69
2013	4.49	2.00
2014	4.36	2.13
2015	4.55	2.44
2016	4.40	2.29
2017	4.63	2.25
2018	4.64	1.91
2019	4.61	2.18

Supplementary Table S8. Age-adjusted incidence rates of ESCC by race/ethnicity: SEER 12 registries between 1992 and 2019.

Year	NHW	NHB	API	Hispanic
1992	2.60	11.99	2.45	3.06
1993	2.39	10.36	3.67	2.70
1994	2.22	10.93	3.04	2.02
1995	2.50	10.21	3.03	2.43

1996	2.26	10.62	3.78	1.85
1997	2.34	9.22	3.39	1.88
1998	2.17	7.99	3.82	1.51
1999	2.13	7.37	3.16	2.48
2000	1.86	6.96	2.88	2.00
2001	2.24	6.05	2.75	1.89
2002	1.83	6.65	2.92	1.62
2003	1.85	7.09	2.04	1.54
2004	2.07	6.17	3.12	1.99
2005	1.78	5.35	2.33	1.59
2006	1.77	5.46	2.69	1.32
2007	1.74	5.03	2.22	1.72
2008	1.62	4.46	2.55	1.32
2009	1.72	5.00	1.93	1.46
2010	1.54	4.63	2.61	1.87
2011	1.62	3.97	1.89	1.29
2012	1.55	3.50	1.82	1.32
2013	1.48	3.77	2.16	1.28
2014	1.41	3.57	1.77	1.35
2015	1.55	3.00	2.10	1.36
2016	1.25	3.31	2.32	1.25
2017	1.46	3.06	2.24	1.06
2018	1.36	3.21	1.68	1.09
2019	1.27	2.06	2.14	1.33
